# Phantom model and scoring system to assess ability in ultrasound-guided chest drain positioning

**DOI:** 10.1186/s13089-016-0038-8

**Published:** 2016-02-18

**Authors:** Luigi Vetrugno, Giovanni Volpicelli, Federico Barbariol, Ilaria Toretti, Livia Pompei, Francesco Forfori, Giorgio Della Rocca

**Affiliations:** Anesthesia and Intensive Care Medicine-Department of Medical and Biological Sciences, University of Udine, P.le S. M. della Misericordia 15, 33100 Udine, Italy; Department of Emergency Medicine, San Luigi Gonzaga University Hospital, Turin, Italy; Anesthesia and Intensive Care Medicine IV, Pisa University Hospital, Pisa, Italy

**Keywords:** Chest tube, Chest drainage, Learning, Training, Simulator, Simulation technology

## Abstract

**Background:**

Chest tube positioning is an invasive procedure associated with potentially serious injuries. In the last few years, we have been running a project directed at developing a practical simulator of a surgical procedure taught on our medical training program. The phantom model reconstructs the pleural anatomy, visible by lung ultrasound, used for the assessed performance of the Seldinger technique. The aim of the present study was to investigate the validity of this simulation technology for assessing residents in anesthesia and intensive care medicine; specifically, their skill in positioning a US-guided chest tube drain was tested using the simulator device. The second aim of the paper was to evaluate the learning curve of our residents over their 5-year study course and validate the phantom scoring system.

**Methods:**

This was a prospective, single-blinded observational study. Participants were recruited from residents in anesthesia and intensive care medicine and divided into two groups: ‘Novice’ and ‘Expert,’ based on the course year attended (years 1, 2, and 3 vs. years 4 and 5, respectively). We asked them to position a chest tube drain in a phantom model, guided by ultrasound, to drain a simulated pleural effusion. Each subject performed two tests that simulated pleural effusions of 4 and 2 cm, respectively. Every step of the maneuver was constantly monitored and the performance scored by the investigators. We then performed a Spearman correlation analysis to evaluate the effect of experience level on the performance of the two groups of residents.

**Results:**

Thirty-one residents were included in this study: 20 in the Novice group and 11 in the Expert group. The mean performance rating score was 0.75 ± 4.38 for the Novice Group and 5.91 ± 3.75 for the Expert group (*p* = 0.0026). The Spearman correlation analysis examining the relationship between year of residency and performance rating score confirmed a positive correlation (*r* = 0.58, *p* = 0.0006). Post-test trend analysis revealed a statistically significant linear trend for skill growth across time, i.e., course year (*p* = 0.0022).

**Conclusions:**

Our simulated procedure using a phantom model of lung anatomy can accurately and reliably be used to assess the skill levels of operators in their ability to drain pleural effusion.

**Electronic supplementary material:**

The online version of this article (doi:10.1186/s13089-016-0038-8) contains supplementary material, which is available to authorized users.

## Background

Percutaneous pleural drain positioning is the third most technical procedure performed in the intensive care unit (ICU) after vascular catheterization and tracheal intubation. Pleural drain positioning can constitute a crucial maneuver for the treatment of critically ill patients [1–3]; for example, it has the potential to improve respiratory function, thereby avoiding intubation in spontaneous breathing patients or leading to earlier weaning in patients under mechanical ventilation. Because of its paramount clinical utility, chest tube positioning has become an incumbent skill for many specialists, such as general surgeons, intensivists, pulmonologists, and emergency medicine specialists [4, 5]. However, pleural drainage is burdened by substantial risks and can result in potentially serious harm if not performed accurately [6, 7]. Even if small-bore tubes are inserted using the Seldinger technique, the procedure is not without risk due to the many major vascular and visceral structures that lay in close proximity to the usual insertion sites and the course of the chest tubes [8]. According to some studies, the complication rate of this procedure ranges from 21 to 30 % [9–11].

Meanwhile, the growing use of point-of-care ultrasound (US) has led to an improvement in the bedside diagnosis and fluid quantification of pleural effusion, and US guidance has proved to increase the success rate of chest tube insertion and the safety of the procedure [12, 13]. International guidelines now recommend US to guide all pleural drainage procedures [14]. This has led to the need for the novice not only to acquire the traditional skills necessary for the Seldinger technique but also to achieve an appropriate level of pulmonary US knowledge. Despite these institutional recommendations, a survey performed at 101 acute hospitals in the UK showed that formal training in the subject matter is not yet sufficiently widespread [6]. In 2010, the British Thoracic Society (BTS) updated its guidelines, recommending that all clinicians performing a chest drain insertion should be appropriately trained and/or supervised [14]. Training should include a theoretical component covering the procedure, pleural anatomy and the risks involved, and a practical component involving a manikin as well as supervised clinical practice. The first aim of our study was to test a new model for assessing the skills of our residents training in anesthesia and intensive care medicine (divided into Novices and Experts) in positioning a US-guided chest tube drain using a simulator device. The second aim was to evaluate the learning curve of our residents over the 5 years of their medical training program and validate the phantom scoring system.

## Methods

### Study setting

This was a prospective, single-blinded observational study for the evaluation of chest drain placement in a US-compatible simulator. Verbal consent was obtained from each participant, and each performance was recorded anonymously. Study subjects were blinded to the purpose of the study and to the parameters that were recorded and scored. Authorization for the study, which did not involve any patients, was given by the Ethical Committee of the Azienda Ospedaliero-Universitaria of Udine. The study was performed in the Department of Anesthesia and Intensive Care Medicine, in the School of Medicine and Residency of the University of Udine, Udine, Italy. Study participants were recruited from residents in anesthesia and intensive care medicine and then divided into two groups: the ‘Novice’ group including residents attending the 1st, 2nd, and 3rd years of the 5-year program and the ‘Expert’ group including residents attending the 4th and 5th years. Our school of residency has an active ultrasound education program that runs throughout the course of the 5 years of residency. As suggested by the American College of Chest Physicians [15], the program is divided into general critical care ultrasonography (GCCUS; which includes vascular and lung US) and critical care echocardiography (CCE). During this training period, residents are required to acquire all of the abovementioned technical skills and are gradually introduced to clinical practice under supervision.

### Phantom model and technique

**Fig. 1:**
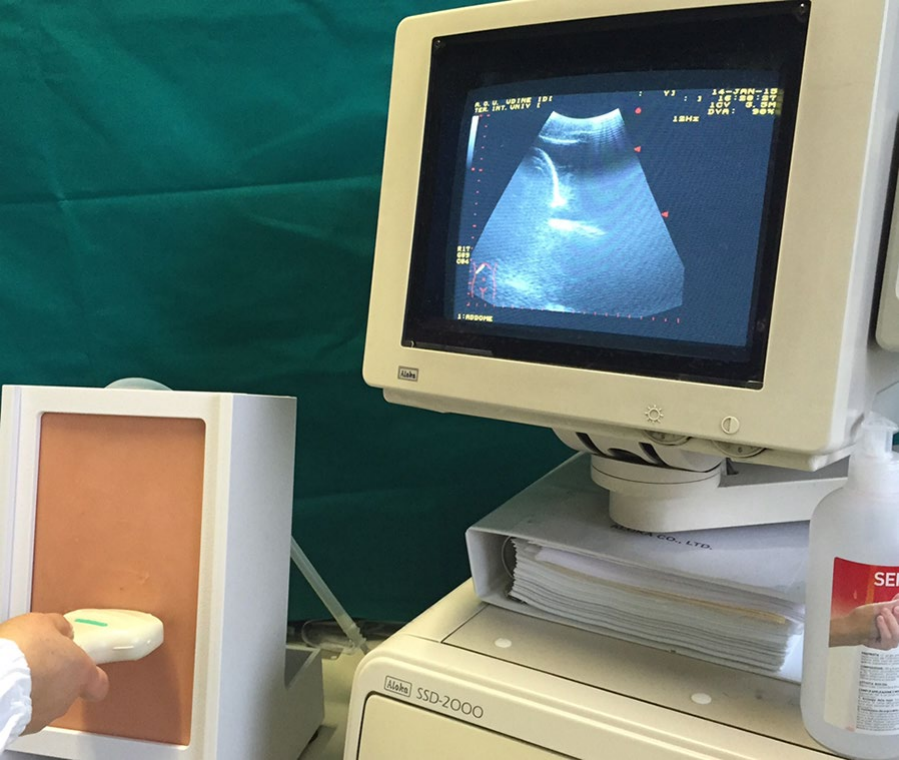
The thoracentesis simulator ‘Ultrasound Thoracentesis Model THM-30’ developed by SIMULAB, Seattle, USA. It features a partial torso with anatomical landmarks, such as skin texture, ribs, and a fluid reservoir. Its simulated lung is seen as an echogenic structure with an inflating mechanism to adjust the size of the pleural effusion

**Fig. 2:**
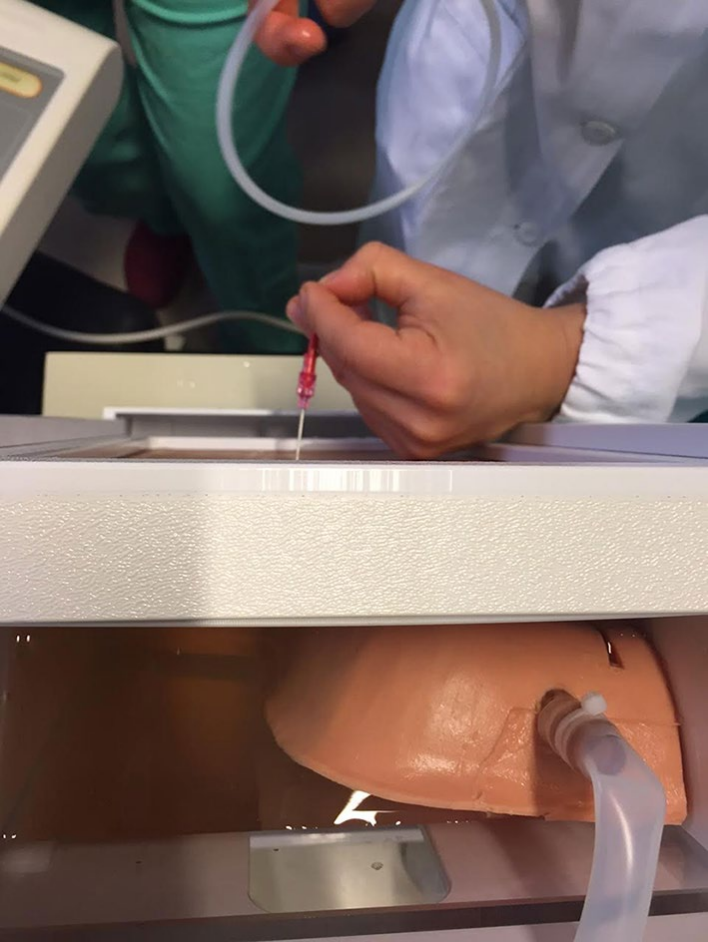
The open top of the Ultrasound Thoracentesis Model. The model’s open top allows the instructor to provide feedback on procedural concepts, offering students the possibility to visualize the catheter depth and placement once inserted into the pleural cavity. This characteristic was used by the investigators to evaluate the performance of each subject

We used the simulator ‘Ultrasound Thoracentesis Model THM-30′ (SIMULAB, Seattle, USA). This simulator features a partial torso with anatomical landmarks such as skin texture, ribs, and a fluid reservoir. Needles and catheters can be inserted and fluid withdrawn (Fig. 1). The simulated lung can be seen as an echogenic structure, and it is equipped with an inflating mechanism that modulates the size of the pleural effusion. It includes a reconstruction of the chest wall tissues, the 6th, 7th, 8th, and 9th ribs with intercostal spaces, the pleural cavity with a normal lung and an atelectatic lung, the diaphragm, and the sub-diaphragmatic spleen. The open configuration of the model (Fig. 2) allows the instructor to provide feedback on procedural concepts, offering students the possibility of visualizing the catheter depth and placement once inserted into the pleural cavity and enabling instructors to evaluate student performance. A positive fluid outflow offers useful positive feedback when pleural effusions are correctly drained.

Each resident was asked to position the needle tip carefully through the phantom manikin, using continuous US guidance to target the pleural effusion. Residents then inserted a Seldinger guidewire into the pleural space, on to which a dilator and then a catheter were subsequently passed to drain the pleural effusion. For this purpose, a 5-Fr pediatric Pneumopericardial Drainage Set (Cook Medical, Bloomington, USA) was used. Two successive tests were performed by each subject: in the first, we simulated a 4 cm pleural effusion (corresponding to about 800 mL) to drain; in the second, the effusion was 2 cm (about 400 mL). No time pressure was put on the study subjects, and they were free to utilize the ultrasound approach they desired. Every step of the manoeuver was constantly observed by the investigators through the opening of the cephalic part of the phantom, and checked for injuries, technical errors, and/or difficulties.

### Scoring system

A 9-item Case Report Form (Additional file 1: CRF) was designed for the investigators’ evaluation of each resident. Each skill or action was scored dichotomously (correct or incorrect). This CRF was then used in a pilot examination of two chief medical residents (FB, IT) in order to estimate the checklist’s reliability and validity. Two assessors scored each resident’s performances: the senior sonographer for our Department (LV) and a trained resident (IT). After each test, the two investigators discussed the case, with the aim of reaching a common assessment. In the case of disagreements, the lowest score was kept.

The specific data collected were the following:

- previous experience: year of residency, number of previous chest drains positioned, and type of US technique used.
- actual performance: success in pleural drainage (at 2 and 4 cm), development of pneumothorax (at 2 and 4 cm), dilator trauma, rib trauma, difficulties (at 2 and 4 cm) with catheter manipulation or placement, and difficulties with the use of US.

Using these data, we calculated a global numerical performance rating score (PRS, ranging from −10 to +10) for the assessment of each subject’s overall performance and ability; all subsequent statistical analyses were performed using these score values. The PRS was calculated as follows:

- evidence of pneumothorax at 4 cm effusion pleural drainage: −3 points. No pneumothorax: +2 points;
- evidence of procedural difficulties at 4 cm effusion pleural drainage: −3 points. No difficulties: +2 points;
- evidence of pneumothorax at 2 cm effusion pleural drainage: −2 points. No pneumothorax: +3 points;
- evidence of procedural difficulties at 2 cm effusion pleural drainage: −2 points. No difficulties: +3 points;

See Additional file 1 for the CRF and PRS calculation. CRF was reviewed for completeness and consistency by authors VL and IT, who frequently perform and supervise thoracentesis.

### Statistical analysis

The data collected for the two groups were first tested for normality using the D’Agostino-Pearson omnibus normality test; descriptive statistics (mean and standard deviation for quantitative variables, and absolute and relative frequencies for qualitative variables) were subsequently calculated for each group. To test for a difference between the two groups with respect to the performance score, we implemented a two-sided unpaired *t* test as well as an *F* test to compare variances and to control whether the *t* test assumptions were met. Our second aim was to assess how performance of the maneuver developed over the 5 years of residency. To this end, we performed a Spearman rank correlation analysis to evaluate the effect of experience level, examining the relationship between year of residency and PRS. We also performed a Pearson correlation (using true values), to see if its result was comparable to that obtained with the Spearman rank correlation, as their difference (or lack thereof) will provide additional information. We subdivided residents according to their year of training and compared their PRS by one-way analysis of variance (ANOVA) and then performed a trend analysis using an ANOVA post-test to test the hypothesis that skill development follows a linear trend according to number of years of experience (i.e., year of residency). Sample size was not calculated before the study given the absence of available comparable data in literature; instead, a retrospective power analysis was implemented. Our goal was to achieve a power of 80 % for a 0.05 significance level (alpha error) using a two-tailed unpaired *t* test.

Statistical analyses were performed using a specifically designed Microsoft Excel 2010 spreadsheet (Microsoft, Redmond, Washington) and GraphPad Prism, version 6.01 for Windows (GraphPad Software, San Diego, USA).

## Results

Thirty-one residents with different skill levels in US-guided techniques and different amounts of previous experience were enrolled in this study. Twenty subjects were assigned to the ‘Novice’ group and 11 to the ‘Expert’ group. The distribution of the study subjects by residency year and the mean performance rating scores per year are shown in Table 1.

**Table 1:** Distribution of study subjects by residency year and mean performance rating scores

Year	*N* (%)	PRS (SD)	Group	*N* (%)	PRS (SD)
1	4 (12.9)	−1.25 (2.5)	Novice	20 (64.5)	0.75 (4.38)
2	8 (25.8)	0.00 (2.67)			
3	8 (25.8)	2.50 (5.98)			
4	5 (16.1)	6.00 (4.18)	Expert	11 (35.5)	5.91 (3.75)
5	6 (19.4)	5.83 (3.76)			
Total	31 (100)	*p* = 0.025		31 (100)	*p* = 0.0026

**Fig. 3:**
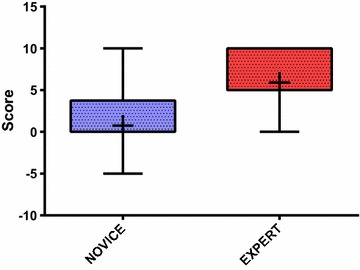
Performance rating score for the Novice and Expert groups. The *colored boxes* extend from the 25th to 75th percentiles. The *whiskers* indicate the minimum and maximum values. The *plus signs* indicate the mean value. *p* = 0.0026

**Fig. 4:**
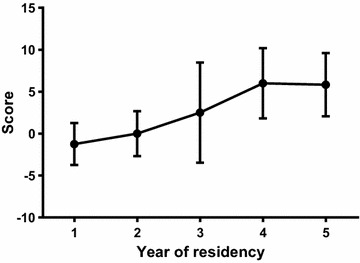
Mean performance rating score in residents subdivided by year. *Error bars* represent standard deviations. The development of residents’ skills in pleural effusion ultrasound and chest drain positioning appears to progress steadily with increasing years of residency, reaching a plateau in the last 2 years. *p* = 0.025

Each resident made two successive attempts at the procedure (making a total number of 62 test attempts for the study group). All of the residents were able to complete the procedure. Data from the two groups were first tested for normality and were found to be well approximated by a normal distribution. With regard to the baseline skills for the two groups, the mean performance rating score was 0.75 ± 4.38 for the Novice Group and 5.91 ± 3.75 for the Expert group (*p* = 0.0026; *F* test to compare variances, *p* = 0.6329; Table 1; Fig. 3), with a difference between means of 5.16 (95 % CI 1.956–8.362). These results confirm a statistically different stratification of participants according to their experience level. Given the difference between the two means and the sample size of our two groups and their standard deviation, the power of the study was higher than 88 % for a significance level (alpha error) of 0.05 with a two-tailed unpaired *t* test, exceeding our a priori power goal of 80 %. Regarding the development of this skill over the 5 years of residency, we performed correlation analyses to evaluate the effect of year of residency. The Spearman correlation analysis examining the relationship between year of residency and PRS confirmed a positive correlation (*r* = 0.58, *p* = 0.0006, 95 % confidence interval 0.2799–0.7820; Table 2), thus proving that higher scores were obtained by residents with greater levels of experience (i.e., years of residency). Correlation analysis was repeated using the Pearson test, and the same results were obtained, further consolidating these results (Pearson *r* = 0.56, *p* = 0.001, 95 % confidence interval 0.2571–0.7633; Table 2). Finally, residents were re-divided according to year of residency and the mean PRSs compared using one-way ANOVA (Table 1; Fig. 4). Once again, a statistically significant different distribution of the mean score was obtained across years (*p* = 0.025). Trend analysis was then performed using an ANOVA post-test and confirmed our hypothesis that a linear trend exists between PRS magnitude and year of residency (*p* = 0.0022—see Fig. 4).

**Table 2:** Correlation analysis examining the relationship between year of residency and performance rating score

Spearman	0.58 (*p* = 0.0006)
Pearson	0.56 (*p* = 0.001)
ANOVA post-test linear trend analysis	*p* = 0.0022

Potential complications resulting from each attempt were also assessed using the phantom dummy (Table 3); the main complication suffered concerns the occurrence of dilator trauma, caused by inserting the dilator too far beyond the pleura, which threatens to cause damage to the lung parenchyma. Dilator trauma occurred in 14.5 % of tests, especially in the Novice group. Other less frequent complications were the development of pneumothorax (defined as accidental puncture of the lung parenchyma; in this case of the phantom lung) in 4.8 % of cases, and the development of rib trauma (1.6 %). No statistically significant differences were found on analyzing the distribution of every single complication or the overall rate of complications between the two groups.

**Table 3:** Main complications occurred

Group	Novice, *n* (%)	Expert, *n* (%)	Total, *n* (%)
Number of tests	40	22	62
Complications			
PNX (at 2 cm only)	2 (5)	1 (4.5)	3 (4.8)
Rib trauma	0 (0)	1 (4.5)	1 (1.6)
Dilator trauma	6 (15)	3 (13.6)	9 (14.5)

## Discussion

The main achievement of this study is to show that the phantom model with the scoring system developed by the authors is useful to assess skills in US pleural puncture and drainage positioning. Another important finding of this study is that residents clearly showed a rapid progression in their proficiency in US-guided pleural drain positioning in a phantom model. This learning curve appears to progress steadily over the 5-year residency course (Fig. 4), with significant improvements evident in the first 3 years, and a plateau from the 4th year onward.

In order to increase patient safety, our institution has designed and implemented a teaching program in which residents are gradually given more responsibility in performing US-guided maneuvers: the first procedures performed by residents are vein and arterial cannulations, followed by loco-regional anesthesia, paracentesis, and finally lung and pleural procedures. Along this learning journey, the use of simulation technology for practicing chest tube positioning provides an important stepping stone before a resident moves on to real patients under a qualified supervision.

Salamonsen et al. [16] assessed 22 physicians (eight novices, seven intermediates and seven advanced) performing thoracic ultrasound on a pleural effusion phantom. These authors found the mean scores for the novice, intermediate, and advanced groups to be 49.3, 73.0, and 91.5, respectively, in a 100-point scale, with a significant difference between groups (*p* < 0.0001). The authors concluded that their phantom procedure could be used to determine the ‘adequacy’ of thoracic ultrasound training before the physician moves into clinical practice, and/or as a tool that provides a way of documenting ongoing procedural competence. Scoring systems for assessing technical competence have already been profitably used and validated in echocardiography too [17, 18].

Wayne et al. [19] reported the use of a thoracentesis model in a structured educational program, giving trainees the opportunity for deliberate practice with feedback, which resulted in consistent improvements in residents’ skills. In agreement with our data, most of their study subjects (who were internal medicine residents) demonstrated poor clinical skill in thoracentesis procedures at the baseline level. It could be argued that some inexperienced participants may have a natural affinity for this procedural task and would be able to develop the required proficiency more rapidly than other more experienced trainees. This was only partially found to be true in our study; we observed a continual growth in US-guided procedure skills with increasing years of residency—comparable to a ‘snowball effect.’ Our teaching program begins with theoretical classes on the basic principles, followed their application in real clinical practice, according to the residency year. For instance, the residents acquire US skills for guided central venous catheterization in their 1st and 2nd years and for nerve blocks in their 2nd and 3rd years. The nature of this curriculum may underlie the linear growth of skill acquisition in thoracic ultrasound and chest tube positioning.

Our study is unique in its field because it combines an assessment of the skills required for lung ultrasound examination with testing for proficiency in performing the Seldinger technique.

The UK National Patient Safety Agency, in the aftermath of 12 deaths and 15 incidents of serious harm following chest drainage procedures over a three-year period (2005–2008), published a warning report [20] which showed that the vast majority of complications were the result of inexperience, inadequate training, and/or poor technical skills; this has since been confirmed by other studies [21, 22]. It is probable that these complications could have been avoided if US guidance had been used. Another important aspect reported by this UK Agency was the lack of familiarity with the Seldinger technique for chest drain insertion and the excessive insertion of the dilator.

In our study, this latter procedural error was indeed the main complication encountered, occurring in 6 out of 40 tests in the novice group (15 %) and in 3 out of 22 tests in the expert group (13.6 %); the overall incidence was 14.5 %. A dilator should never be inserted by more than one centimeter, as recommended by the BTS guidelines [14]. We also observed 3 cases of pneumothorax, all of which occurred when the pleural effusion was 2 cm (2 cases in the Novice group and 1 in the Expert group), while no cases of pneumothorax were observed when the pleural effusion was 4 cm. The most obvious explanation for this is the larger safety margin when the extent of pleural effusion is greater. Finally, a single case of a broken needle also occurred, caused by impact against a phantom rib. According to our teaching program, residents in their 4th and 5th years should achieve the necessary levels of proficiency in lung ultrasound to distinguish all the thoracic anatomic structures and to identify pleural effusion. This is critical for guaranteeing basic levels of competence and safety in thoracic procedures. In our opinion, thoracic ultrasound should be practiced daily in the ICU to ensure the progressive improvement of these fundamental skills.

## Limitations

This study has a number of limitations. First, it was conducted in a single teaching hospital in a selected population of trainee physicians (residents in anesthesia and intensive care medicine). For this reason, our results cannot be extended to other academic institutions. The phantom model that we used is a thoracentesis model adapted to pleural chest drainage performed using small 5-French pediatric pig-tail catheters. This device is not commonly used as a chest drain and presents some limitations. For example, penetration of the phantom’s gel layers becomes more difficult after a failed first attempt, because the catheter gradually warms up and loses its rigidity, which makes all further attempts more difficult. Another limitation of our study is that the investigators rated the residents’ performances according to their own overall impression of each subject’s ability, without the application of any objective assessment criteria. We did, however, try to limit any potential bias by combining a double evaluation on the same performance, released by two independent investigators, one of whom was the senior sonographer of our department.

Simulation technology could be used to document the progression of technical proficiency during residency training and to determine the adequacy of a doctor’s mastery of the procedure for chest drainage before their introduction into clinical practice. However, working on a simulator manikin is less technically challenging and stressful compared with performing the same procedure on real patients. A physician’s expertise must include the ability to communicate with the patient, to adapt techniques to different body habitus and to tailor the procedure to specific situations. None of these can be currently trained using simulator technology. Simulation-based training provides a relaxed environment where technical skills can be practiced, but it cannot completely substitute the real clinical setting. Finally, another limitation of our model is that a phantom simulator cannot test for the occurrence of a hemothorax.

## Conclusion

Our phantom model and scoring system provide a reliable method for the assessment of procedural competences in ultrasound and pleural drain positioning. Traditionally, the teaching model for medical education has primarily been based on direct experiential learning in the clinical environment. However, a more contemporary concept of training should also include theoretical components as well as the opportunity for the simulated practice of supervised procedures using suitable technology before proceeding on to real patients.

## Additional file

10.1186/s13089-016-0038-8 A 9-item Case Report Form.

## Abbreviations

ANOVA: analysis of variance
BTS: British Thoracic Society
CCE: critical care echocardiography
GCCUS: general critical care ultrasonography
ICU: intensive care unit
NPSA: National Patient Safety Agency
PRS: performance rating score
US: ultrasound
