# Supplementary material for: Phantom model and scoring system to assess ability in ultrasound-guided chest drain positioning
Source: Crit Ultrasound J. 2016 Feb 18;8:1. doi: 10.1186/s13089-016-0038-8 (PMC4759451; doi:10.1186/s13089-016-0038-8)
Supplement: Supplementary file 1 — 10.1186/s13089-016-0038-8 A 9-item Case Report Form. [file 13089_2016_38_MOESM1_ESM.docx]

**CRF PHANTOM STUDY**

**TEST SUBJECT NUMBER:**

| **PRE EXPERIENCE**: |  |  |
| --- | --- | --- |
| - YEAR OF RESIDENCY |  |  |
| - NUMBER OF CHEST DRAINS ALREADY POSITIONED |  |  |
| - TYPE OF USED US TECHNIQUE: |  |  |
|  |  |  |
| **PERFORMANCE EVAL:** |  | ***SCORE*** |
| - 4 CM PLEURAL DRAINAGE |  | 0 |
| - 4 CM PNX |  | -3 +2 |
| - 4 CM DIFFICULTY |  | -3 +2 |
| - - CATHETER DIFFICULTY   - US DIFFICULTY |  |  |
| - 2 CM PLEURAL DRAINAGE |  | 0 |
| - 2 CM PNX |  | -2 +3 |
| - 2 CM DIFFICULTY |  | -2 +3 |
| - - CATHETER DIFFICULTY   - US DIFFICULTY |  |  |
| - DILATOR TRAUMA |  |  |
|  |  |  |
|  |  | ***TOTAL:*** |
|  |  |  |
